# Enhancing the Biosorption Capacity of *Macrocystis pyrifera*: Effects of Acid and Alkali Pretreatments on Recalcitrant Organic Pollutants Removal

**DOI:** 10.3390/ijms26073307

**Published:** 2025-04-02

**Authors:** Magdalena Varas, Jorge Castro-Rojas, Loretto Contreras-Porcia, María Soledad Ureta-Zañartu, Elodie Blanco, Néstor Escalona, Edmundo Muñoz, Elizabeth Garrido-Ramírez

**Affiliations:** 1 Escuela de Ciencias Ambientales y Sustentabilidad, Universidad Andres Bello, República 440, Santiago 8370251, Chilej.castrorojas3@uandresbello.edu (J.C.-R.); 2 Departamento de Ecología y Biodiversidad, Facultad Ciencias de la Vida, Universidad Andres Bello, República 440, Santiago 8370251, Chile; lorettocontreras@unab.cl; 3 Centro de Investigación Marina Quintay (CIMARQ), Facultad Ciencias de la Vida, Universidad Andres Bello, Quintay 2531015, Chile; 4 Center of Applied Ecology and Sustainability (CAPES), Santiago 8331150, Chile; 5 Instituto Milenio en Socio-Ecología Costera (SECOS), Santiago 8370251, Chile; 6 Departamento de Ciencias del Ambiente, Facultad de Química y Biología, Universidad de Santiago de Chile, Santiago 9170022, Chile; soledad.ureta@usach.cl; 7 Departamento de Ingeniería y Gestión de Construcción, Escuela de Ingeniería, Pontificia Universidad Católica de Chile, Avenida Vicuña Mackenna 4860, Macul, Santiago 7820436, Chile; elblanco@uc.cl; 8 ANID–Millennium Science Initiative Program, Millennium Nuclei on Catalytic Process towards Sustainable Chemistry (CSC), Santiago 7820436, Chile; 9 Departamento de Ingeniería Química y Bioprocesos, Escuela de Ingeniería, Pontificia Universidad Católica de Chile, Avenida Vicuña Mackenna 4860, Macul, Santiago 7820436, Chile; 10 Centro de Investigación para la Sustentabilidad (CIS), Facultad Ciencias de La Vida, Universidad Andres Bello, República 440, Santiago 8370251, Chile; edmundo.munoz@unab.cl

**Keywords:** sustainable biosorption, *Macrocystis pyrifera*, recalcitrant organic pollutants, wastewater treatment, water purification technologies

## Abstract

The effects of acid and alkali pretreatments on the physicochemical and textural properties of *Macrocystis pyrifera* were evaluated to assess its potential for removing recalcitrant organic pollutants from aquatic systems. Untreated (UB), acid-pretreated (A_C_PB), and alkali-pretreated (A_L_PB) seaweed biomass were characterized using SEM, FTIR-ATR, N_2_ adsorption–desorption, and potentiometric titrations. Adsorption isotherms and kinetic studies, using methylene blue (MB) as a model pollutant, were conducted to evaluate removal performance. All biosorbents exhibited Langmuir behavior, with maximum adsorption capacities of 333 mg g^−1^ (UB), 189 mg g^−1^ (A_C_PB), and 526 mg g^−1^ (A_L_PB). FTIR-ATR and SEM analyses revealed that alkali pretreatment increased the abundance of hydroxyl, carboxylate, and sulfonated functional groups on the seaweed cell walls, along with greater porosity and surface roughness, resulting in enhanced MB adsorption. In contrast, acid pretreatment increased the exposure of carboxylic, amine, and amide functional groups, reducing the electrostatic interactions. The adsorption energy values further supported this, while the intra-particle diffusion model indicated a two-step process involving MB diffusion onto the seaweed surface, followed by diffusion into internal pores. These findings highlight the potential application of *Macrocystis pyrifera*-based biosorbents in the treatment of wastewater containing recalcitrant organic pollutants.

## 1. Introduction

Studies reporting the presence of recalcitrant organic pollutants in aquatic systems have increased in recent years [1,2,3]. Recalcitrant organic pollutant removal by conventional wastewater treatments is difficult due to its toxicity and high chemical stability [3]. The abatement of these compounds from wastewater is crucial to protect human health and the environment and aligns with the targets of United Nations Sustainable Development Goal 6 (ODS 6), which emphasizes ensuring the availability and sustainable management of water and sanitation for all (reuse and sustainable use of wastewater by 2030) [4]. Achieving this goal requires the development and implementation of suitable, low-cost, and environmentally friendly technologies. In this sense, the biosorption process appears to be a promising alternative, either alone [5,6] or combined with advanced oxidation processes to concentrate the organic pollutant for further oxidation [7,8,9,10,11].

Biosorption is defined as the capacity to sequester compounds by live or dead biomass derived from industrial or agricultural by-products, forestry, marine or terrestrial biological materials, and microbe biomass [12,13]. Among biosorbents, seaweeds have comparative advantages over other biomass types because they are abundant in marine environments, can be reused, and have a high pollutants adsorption capacity [14]. This high adsorption capacity to binding pollutants has been attributed to the presence of polysaccharides, proteins, and lipids on the cell wall surface containing different functional groups, such as amino, hydroxyl, carboxyl, and sulfonate groups, among others [14,15].

Studies on the use of seaweeds as biosorbents have shown an enhancement in the pollutant adsorption capacity if the biomass is chemically pretreated (washing) with different solutions, such as formaldehyde, ethyl alcohol, acetone, calcium chloride, alkali, and acid, among others [5,16,17,18]. This method is preferred due to its simplicity and efficiency, especially when a low concentration of acid or alkali solutions is employed [16]. However, in the case of chemical pretreatment with acid or alkali solutions, different effects in the sorption process have been reported, depending on the seaweed used and the kind of pollutant [5,19]. By using acid or alkali pretreatment, different functional groups present on the seaweed wall surface can be newly formed, increasing their amount or even decreasing it, thus affecting the pollutant biosorption favorably or unfavorably [16]. For example, an enhancement in the biosorption capacity of phenoxy alkanoic acid herbicide (2,4-D) and Cr(VI) was reported for *Gracilaria verrucosa* biomass pretreated with acids, whereas the alkali pretreatment led to a reduction in the adsorption of both herbicide and metal [16]. Contrary to this, Daneshvar et al. [5] showed a decrease in methylene blue adsorption for brown (*Nizamuddinia zanardinii*) and green (*Ulva fasciata)* algae pretreated with acid, whereas no significant effect was observed on red alga (*Gracilaria parvispora*) biosorption capacity. When the algae were pretreated with alkali solution, the methylene blue adsorption enhanced significantly for red and green algae, but the biosorption of brown algae was decreased [5]. Therefore, studying the effect of chemical pretreatment with both acid and alkali solutions on the surface properties and adsorption capacity of seaweed is a crucial prerequisite for selecting a particular organism for the removal of organic pollutants.

*Macrocystis pyrifera* (*M. pyrifera*) is a brown seaweed frequently found in coastal regions of Mexico, the United States, Peru, Chile, Argentina, South Africa, Australia, New Zealand and the Sub-Antarctic Islands [20,21]. *M. pyrifera* has extremely high fertility and can achieve greater biomass compared to other species [21]. Therefore, it exhibits great potential to be used as a biosorbent material; indeed, it is widely reported as a biosorbent of metals [17,22,23,24] and used to produce biochar [25]. However, less information is available about its use for organic pollutants [26], while, as far as we know, the biosorption capacity of recalcitrant organic pollutants of *M. pyrifera* pretreated with acid or alkali has not been reported.

**Figure 1:**
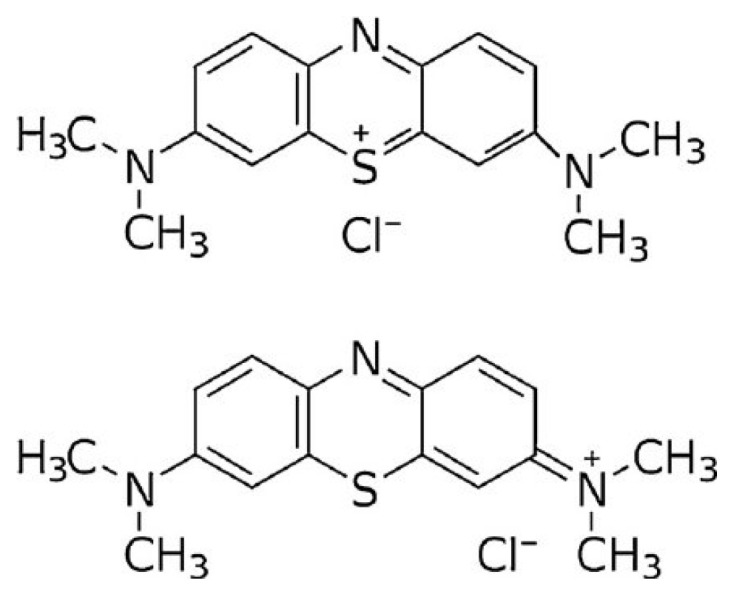
Methylene blue molecule and its resonant structure.

Therefore, this work aimed to study the effect of acid and alkali pretreatment on the biosorption capacity of *M. pyrifera* to remove recalcitrant organic pollutants from an aqueous solution, as well as its relationship with the physicochemical and structural properties of the seaweed biomass. Methylene blue (MB) was chosen as a model molecule of recalcitrant organic pollutants because it is an organic cationic dye widely used in the textile industry and biotechnological applications [27,28]. Its resonant structure is presented in Figure 1, demonstrating that the positive charge can be located on one of the amino nitrogen atoms or the sulfur (S) atom. Some characteristics of MB are presented in Table 1 [29]. The performance of *M. pyrifera* modified with acid and alkali was studied under different pH values and biosorbent dosages. Kinetic studies were also incorporated to study the adsorption mechanism involved in MB biosorption of *M. pyrifera*.

## 2. Results and Discussions

### 2.1. Characterization of M. pyrifera Biomass

#### 2.1.1. SEM and Textural Properties

**Figure 2:**
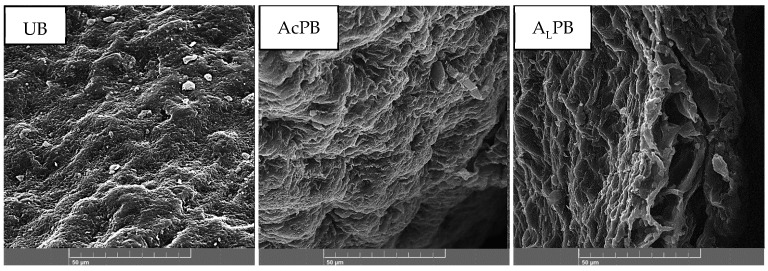
Representative SEM images of untreated (UB), acid-pretreated (A_C_PB), and alkali-pretreated (A_L_PB) seaweed biomass.

SEM images of untreated biomass (UB), acid-pretreated biomass (A_C_PB), and alkali-pretreated biomass (A_L_PB) are presented in Figure 2, showing structural modifications in the morphology of the adsorbent material. The SEM image of untreated biomass exhibits a smoother structure with low roughness and minimal porosity. In the case of acid treatment, a more compact structure is observed, while the case of alkali treatment presents a rougher and more porose morphology compared to UB and A_C_PB. These results suggest that alkali pretreatment induces an opening of the original structure, promoting porosity development.

The SEM images are supported by the BET analysis. The BET surface areas of the three biomass samples (Table 2) are lower than 1.0 m^2^ g^−1^, similar to previous studies reporting low BET surface area for *M. pyrifera* [26,30]. A_C_PB exhibited a 38% reduction in BET surface area compared with UB, accompanied by a decrease in both pore volume and pore diameter (Table 2). In contrast, A_L_PB showed an approximately 60% increase in BET surface area, along with an increase in average pore volume (from 0.0021 cm^3^ g^−1^ to 0.0065 cm^3^ g^−1^) and a reduction in pore diameter (from 114 nm to 86 nm). Thus, alkali pretreatment promotes a reduction of the macropores to mesopores and reduces the size of some mesopores (insets of Figure S1). Consequently, although the average pore diameter decreases, the total pore volume increases due to the formation of additional accessible adsorption sites, in agreement with the observed increase in BET surface area.

Consistently, UB and A_C_PB biosorbents exhibit a Type II isotherm with minimal hysteresis, characteristics of low-porosity materials primarily composed of macropores. A small H3 hysteresis loop is present, indicating the contribution of some slit-like mesopores (Figure S1). A_L_PB biomass presents a Type IV isotherm with an H4 hysteresis loop, characteristics of mesoporous materials [31,32]. The H4 hysteresis loop is typically associated with narrow slit-like pores containing internal voids of irregular shape and a broad size distribution. Thus, the decrease in both macropore and mesopore size, combined with the increase in surface area, confirms that alkali pretreatment reduces the size of existing macro and mesopores.

#### 2.1.2. FTIR Characterization

**Figure 3:**
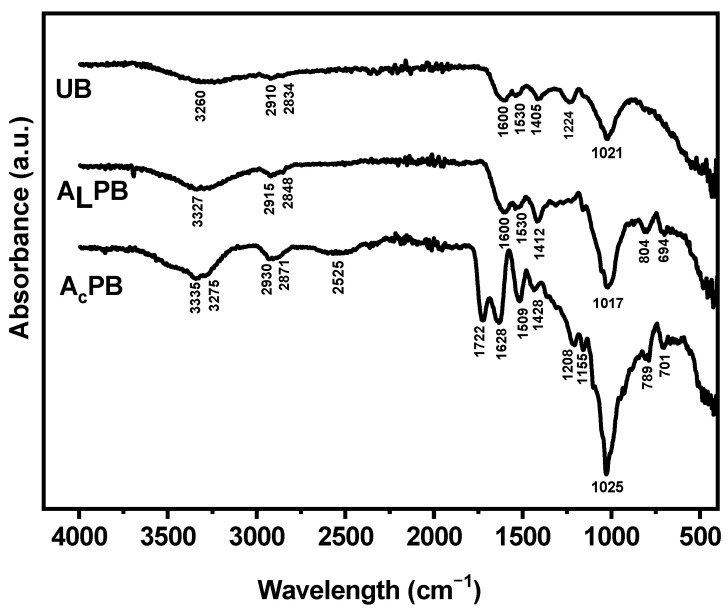
FTIR-ATR spectra for untreated biomass (UB), alkali-pretreated biomass (A_L_PB) and acid-pretreated biomass (A_C_PB).

Figure 3 presents the FTIR-ATR spectra for UB, A_L_PB, and A_C_PB seaweeds. Typical cell walls of *M. pyrifera* comprise a fibrillar structure mainly of cellulose and an embedding matrix of alginic acid or alginate (phyco-colloids) with a small amount of sulfated polysaccharides (fucoidans). Additionally, proteins and lipidic components are present, contributing to the overall chemical functionality of the biomass [24,26,30,33]. These compounds have active groups such as amine, amide, carboxyl, hydroxyl, sulfhydryl, and sulfonate groups that are responsible for the adsorption process [17,23,24]. The FTIR-ATR spectrum of UB shows a broad band at 3260 cm^−1^, attributed to N-H stretching vibrations of amino groups (-NH_2_) and O-H stretching vibrations of hydroxyl groups (-OH), which can be associated with proteins (amino acids) and polysaccharides, respectively [22,24,26,33,34]. The bands at 2910 cm^−1^ and 2834 cm^−1^ correspond to C-H stretching vibrations, with the 2910 cm^−1^ band primarily attributed to carbohydrates in cellulose and hemicellulose, while the 2834 cm^−1^ band is associated with C-H stretching in aliphatic chains of lipids [24,26]. The bands at 1600 cm^−1^ and 1405 cm^−1^ are attributed to C=O stretching of carboxylic acids [35] and symmetric stretching of carboxylate anions (-COO^−^) [33,36], respectively. Additionally, secondary amines (N-H and C-N stretching) are detected at 1530 cm^−1^ [36,37]. The band at 1224 cm^−1^ is attributed to aryl-O stretching aromatic ethers [5]. The band at 1021 cm^−1^ corresponds to the C-N stretching of amine groups and C-O stretching vibration of hydroxyl groups from cellulose and hemicellulose [22,24,26,33,38]. Furthermore, sulfonate groups (-SO_3_^−^) are detected in the range of 700-900 cm^−1^, assigned to S=O and S-O stretching vibrations [17,23].

The alkali and acid pretreatment modified the wall surface of *M. pyrifera*, represented by a shift in the intensity and displacement of some typical bands, besides the presence of new signals compared with untreated biomass (Figure 3). In A_L_PB, the band corresponding to amine and hydroxyl groups shifted from 3260 cm^−1^ to 3327 cm^−1^, being more intense than UB. Similarly, a more pronounced C-H stretching vibration was observed at 2915 cm^−1^ and 2848 cm^−1^, suggesting a higher exposition of lipid-associated structures due to a possible disruption of the cell wall. No significant changes were observed at 1530 cm^−1^, suggesting that secondary amines were not substantially affected by the alkali pretreatment. The increase in intensity at 3327 cm^−1^ could be attributed to a greater availability of hydroxyl groups on the seaweed surface, resulting from the disruption of the polysaccharide matrix during the alkali pretreatment. This assumption is further supported by the increased intensity and slight shift in the C-O stretching band of hydroxyl groups, which shifted from 1021 cm^−1^ to 1017 cm^−1^, indicating partial dissolution of cellulose and hemicellulose. This process enhances porosity and increases the accessibility of adsorption sites, as was confirmed by SEM and surface characterization. Regarding carboxyl (-COOH) and carboxylate (-COO^−^) groups, the most notable difference was observed in the band at 1405 cm^−1^, which became more intense and shifted to 1412 cm^−1^, suggesting an increase in carboxylate anions due to alkali treatment. Conversely, the band at 1600 cm^−1^ remains almost constant. Additionally, new bands were observed in the fingerprint region of the spectrum at 804 cm^−1^ and 694 cm^−1^, attributed to the stretching vibration of S-O and S=O bonds, respectively. These signals are associated with sulfonates groups (-SO_3_), mainly presents in the sulfated polysaccharides (fucoidans) of brown seaweed [24,39,40]. These results are consistent with studies reporting that organic matter in seaweed can degraded during alkaline hydrolysis, leading to the exposure of hydroxyl (-OH) and sulfonates (-SO_3_) groups, as well as the deprotonation of carboxylic (-COOH) into carboxylate (-COO-) functional groups [5].

In the case of A_C_PB, the FTIR-ATR showed a significant difference in comparison with UB and A_L_PB, suggesting that the functional groups on the wall of *M. pryrifera* were substantially modified by the acid pretreatment with sulfuric acid. The increase in intensity and shift in the N-H and O-H stretching bands (from 3260 cm^−1^ to 3335 cm^−1^) suggest an enhanced exposure of amine and hydroxyl groups, likely due to the hydrolysis of polysaccharides and proteins in the seaweed matrix. The appearance of a shoulder at 3275 cm^−1^, attributed to the presence of amine groups, typically masked by the intense vibration of hydroxyl groups, suggest partial degradation of polysaccharides and the cleavage of peptide bonds in proteins [24]. The signals associated with C-H stretching vibrations were more intense than those in UB and A_L_PB and shifted to higher wavelengths (2930 cm^−1^ and 2871 cm^−1^), suggesting increased exposure of lipid-associated structures. New peaks were observed at 2555 cm^−1^ and 1727 cm^−1^, which can be assigned to the overlapping of –OH in the carboxyl group and C=O stretching vibration present in ketones, aldehydes, esters, or carboxylic acid, respectively [41,42]. In addition, new and more intense bands associated with amide and amine groups appear at 1628 cm^−1^ and 1509 cm^−1^ [22,39]. These findings indicate that acid pretreatment leads to the hydrolysis of polysaccharides and proteins, enhancing the exposure of carboxylic acid (-COOH), amine (-NH_2_), and amide (C=O and N-H) functional groups on the biomass surface [41].

#### 2.1.3. Potentiometric Titrations

Table 3 presents the pH value for the point of zero charge (PZC) and intrinsic acid dissociation constants (pKa_1_, pKa_2_, and pKa_3_) determined by the simple extrapolation method, whereas the potentiometric titrations performed at different ionic strength solutions are shown in Supplementary Materials (Figure S2). As expected, the acid and alkali pretreatment modified the surface charge properties of *M. pyrifera*, attributed to the exposure and modification of surface functional groups as was evidenced by the FTIR-ATR analysis.

The PZC of UB was near to 7.1, indicating that under natural conditions, *M. pyrifera* presents a balance between acidic and basic functional groups. After acid pretreatment, the PZC decreased to 5.1, attributed to the contribution of acidic groups due to the increased exposition of amines, hydroxyls, and carboxyl groups as a result of acid hydrolysis of polysaccharides and proteins, as was evidenced by the FTIR-ATR analysis. The shift in pka_1_ from 5.64 (UB) to 4.24 (A_C_PB) suggests a stronger acidic character after acid treatment, while the reduction in pka_2_ from 8.20 to 6.38 is a consequence of modifications in weakly acidic sites.

Similarly, alkali pretreatment exhibited a lower PZC (5.4), attributed to the deprotonation of carboxyl to carboxylate and the higher exposure of sulfonates groups. The increase in pka_2_ from 8.20 to 10.82 suggests modifications in weakly acidic functional groups, potentially associated with newly exposed oxygen-containing groups resulting from alkaline hydrolysis. The pka_3_ values (Table 3) suggest that amine groups were partially retained in A_L_PB but protonated or degraded in A_C_PB [26]. These changes in acidity and surface charge suggest that acid-treated biomass may exhibit a higher affinity for anionic dyes due to its increased acidity, while alkali-treated biomass could favor the adsorption of cationic dyes due to the greater presence of deprotonated groups.

### 2.2. Methylene Blue Biosorption Capacity

#### Adsorption Model

**Figure 4:**
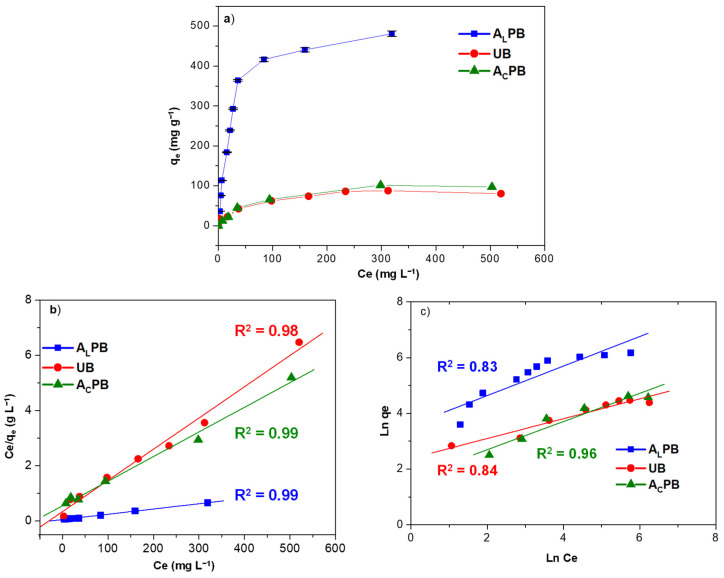
Effect of pretreatment of *M. pyrifera* in the biosorption of methylene blue at room temperature and pH 7.9: (**a**) adsorption isotherms; (**b**) fit of the experimental data to Langmuir isotherm model; (**c**) fit of the experimental data to Freundlich isotherm model.

The effect of acid and alkali pretreatment on the MB adsorption capacity of *M. pyrifera* is presented in Figure 4 and Table 4. The adsorption isotherms (Figure 4a) indicate that the process follows the Langmuir model, with A_L_PB exhibiting the highest saturation capacity, as confirmed by Equation (3). The correlation coefficient (R^2^) indicates that the experimental data fit better with the Langmuir model than the Freundlich model (Table 4), suggesting monolayer adsorption on well-defined active sites. The Langmuir model assumes a homogeneous surface with identical binding sites, whereas biomass-derived adsorbents, such as seaweed, possess a heterogeneous surface with diverse functional groups, as confirmed by FTIR-ATR analysis. While the Langmuir model effectively describes the overall adsorption trends, it does not fully account for surface heterogeneity. To further elucidate the adsorption mechanism, the Dubinin–Radushkevich (D-R) model was also applied.

The D-R results suggest that the adsorption process is primarily governed by a combination of physical and chemical interactions, with a tendency toward chemisorption. The dominant mechanism is likely through electrostatic attraction between the negatively charged functional groups of *M. Pyrifera* and the cationic MB dye. This conclusion is supported by the mean free energy of adsorption (*E*) values estimated from Equation (8), which ranged from 8.45 to 10 kJ mol^−1^ (Table 4). While electrostatic interactions seem to play a crucial role, the potential contribution of ion exchange cannot be entirely dismissed, given the presence of functional groups such as carboxylates and sulfonates, which are known to participate in ion-exchange processes [16,17,40]. However, this study did not specifically assess ion-exchange mechanisms, and further research, including desorption and competitive ion studies, would be necessary to fully elucidate its role in MB adsorption onto *M. pyrifera*.

The alkali pretreatment of *M. pyrifera* increased (by 58%) the maximum adsorption capacity (*Q*_0_) of methylene blue at pH 7.9 from 333 mg g^−1^ to 526 mg g^−1^ (Table 4). In the case of acid pretreatment, the maximum adsorption capacity decreased by 43% (from 333 mg g^−1^ to 189 mg g^−1^). The different performance in terms of the ability to uptake MB from aqueous solution of natural or pretreated *M. pyrifera* can be a consequence of both structure and chemical composition. Structure refers to the specific surface area, porosity, and swilling effects, among others, whereas chemical composition is associated with different functional groups present on the cell wall acting as active sites for binding of the target molecule [30].

The structural modifications induced by alkaline treatment enhance the adsorption capacity of MB by increasing porosity and surface roughness in A_L_PB, which facilitates dye adsorption. In contrast, A_C_PB exhibited a smaller surface area, a more compact structure, and minimal porosity, leading to a lower adsorption capacity, as expected.

Additionally, according to FTIR-ATR data, pretreatment of *M. pyrifera* with acid or alkali modified the functional groups present in the seaweed wall. In A_L_PB, the adsorption process was favored by the presence of hydroxyl (-OH), carboxylate (-COO^−^), and sulfonated (SO_3_^−^) groups, which increased the number of negatively charged adsorption sites. These modifications enhanced electrostatic interactions with positively charged MB molecules, resulting in a higher adsorption efficiency [5]. Furthermore, the increased surface porosity further contributed to greater accessibility of adsorption sites.

On the other hand, acid pretreatment enhancing the exposition of carboxylic (-COOH), amine (-NH_2_), and amide (C=O, N-H) functional groups, altering the surface charge of biomass from slightly negative to slightly positive [13,43]. Consequently, the reduced availability of negatively charged functional groups weakened the electrostatic interactions between the biomass and MB molecules, decreasing the dye’s adsorption capacity.

The maximum MB adsorption capacity obtained in this study was compared with different sorbents reported in the literature (Table 5). A direct comparison is difficult since the experiments were not all conducted under the same experimental conditions. However, alkali-pretreated *M. pyrifera* exhibits a higher MB adsorption capacity compared to some algal species and other adsorbents. The equilibrium parameter (*R_L_*) was determined by using Equation (4) (Table 4). The *R_L_* value indicates that the adsorption of MB by UB, A_L_PB, and A_C_PB is favorable.

### 2.3. Adsorption Kinetic Study

Kinetic models, including pseudo-first order and pseudo-second-order models, were applied to fit the experimental data. Additionally, diffusion-based models such as liquid-film diffusion and intra-particle diffusion were used to predict the dynamic character of adsorption of MB on A_L_PB [45]. The effects of pH and biosorbent dosage in the adsorption kinetics were studied.

**Figure 5:**
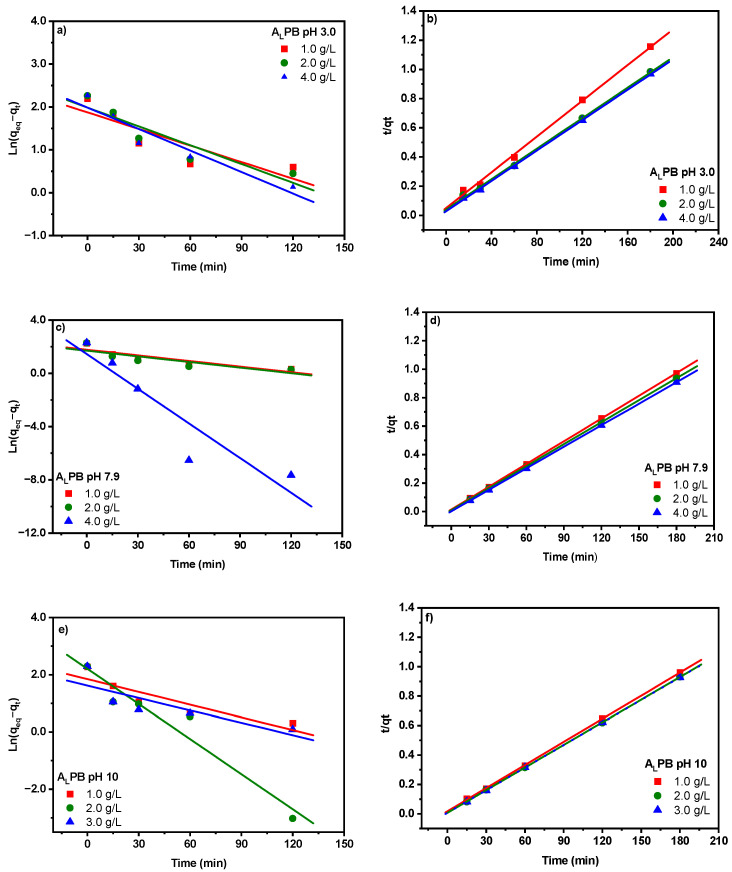
The kinetic of biosorption of methylene blue by alkali-pretreated biomass (A_L_PB) performed at room temperature, different pHs, and different biomass dosages: (**a**,**c**,**e**) pseudo-first-order model kinetic and (**b**,**d**,**f**) pseudo-second-order kinetic model.

The fit of experimental data of MB adsorption on A_L_PB to pseudo-first-order and pseudo-second-order models are presented in Figure 5, and the calculated kinetic parameters are reported in Table 6. For all pH values and dosages studied, the pseudo-first-order model presents a correlation coefficient (R^2^) in the range of 0.637–0.915, which is lower than those obtained by the pseudo-second-order model (Figure 5b,d,f). Therefore, the pseudo-first-order reaction cannot provide accurate experimental data fit. In the case of the equilibrium adsorption capacity determined by the pseudo-second-order model, it was more suitable to experimental values than that calculated with the pseudo-first-order reaction model. These results suggest that the rate-determining step (RDS) of adsorption is the chemical sorption process between MB and functional groups (e.g., -OH, -COO^−^ and SO_3_^−^) present in the alkali-pretreated *M. pyrifera* [5]. While the increase in the pseudo-second-rate constant (*k*_2_) with biosorbent dosage is expected due to the availability of more binding sites, these results highlight the role of alkali pretreatment in enhancing site accessibility and surface charge, leading to improved adsorption efficiency. This finding is particularly relevant in optimizing chemically modified biosorbents for dye removal applications.

**Figure 6:**
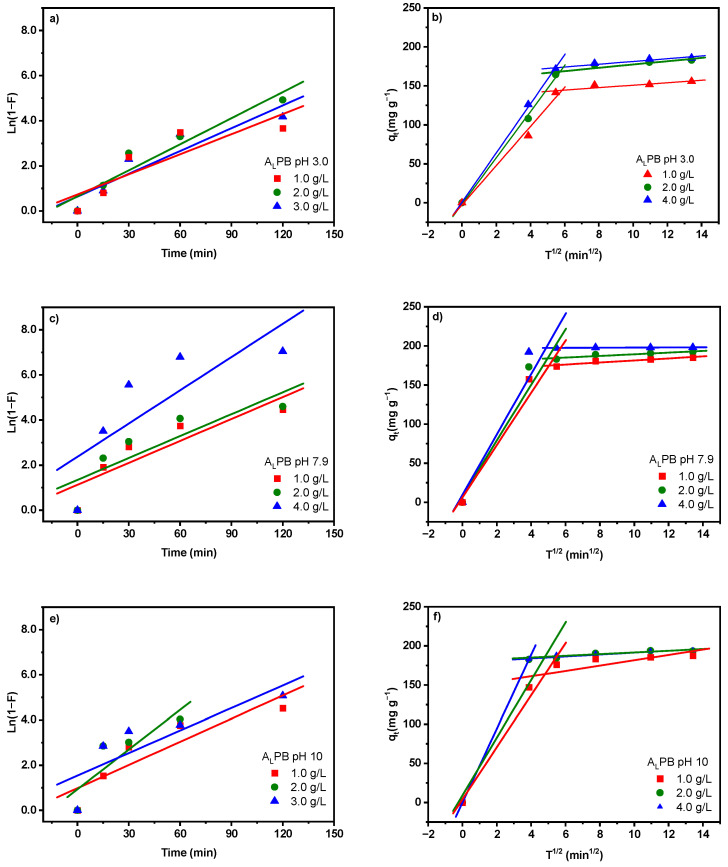
Liquid-film diffusion (**a**,**c**,**e**) and intraparticle diffusion models (**b**,**d**,**f**) of methylene blue biosorption by alkali-pretreated biomass (A_L_PB) performed at different pHs and 1.0 g L^−1^ of biomass dosage.

The fit of experimental data to the liquid-film diffusion and intra-particle diffusion models, performed at different pH values and biosorbent dosages for alkali-pretreated *M. pyrifera*, is presented in Figure 6, and the calculated kinetic parameters are summarized in Table 6. The liquid-film diffusion model suggests that film diffusion is involved in the sorption process if the plot -*Ln*(1 − *F*) vs. *t* is linear, and it is the rate-limiting step if the line passes through the origin [46]. As shown in Figure 6a,c,e and Table 6, the liquid-film diffusion plots exhibit relatively low correlation coefficient (R^2^ values ranging from 0.637 to 0.915) for all pH levels and biosorbent dosages studied. Additionally, the lines do not pass through the origin, indicating that while liquid-film diffusion plays a role in the adsorption process, it is not the rate-determining step in this system. Instead, another mechanism appears to control the overall adsorption kinetics. The intra-particle diffusion model suggests that intra-particle diffusion is involved in the sorption process if the plot *q* vs. *t^1/2^* is linear and is the rate-limiting step where the line passes through the origin [46]. The intra-particle diffusion plots exhibit multi-linearity, as shown in Figure 6b,d,f, where two distinct linear regions with different slope values are observed, suggesting that the biosorption process follows two sequential phases. The calculated intra-particle diffusion coefficients (*K*_*dif*(1)_ and *K*_*dif*(2)_) for these two phases are presented in Table 6.

These results indicate that the biosorption of MB on A_L_PB involves an initial rapid surface biosorption phase, where film diffusion facilitates mass transfer to the adsorbent surface, followed by a slower intra-particle diffusion phase as MB molecules penetrate deeper into the adsorbent structure. Although liquid-film diffusion plays a role, particularly in systems with high concentration gradients [13], the kinetic data suggests that intra-particle diffusion is the rate-limiting step in this system. These results are consistent with the reports in the literature for organic pollutant adsorption by seaweed used as biosorbent [6], in which the biphasic nature of intraparticle diffusion plots has been observed.

### 2.4. Desorption Study

**Figure 7:**
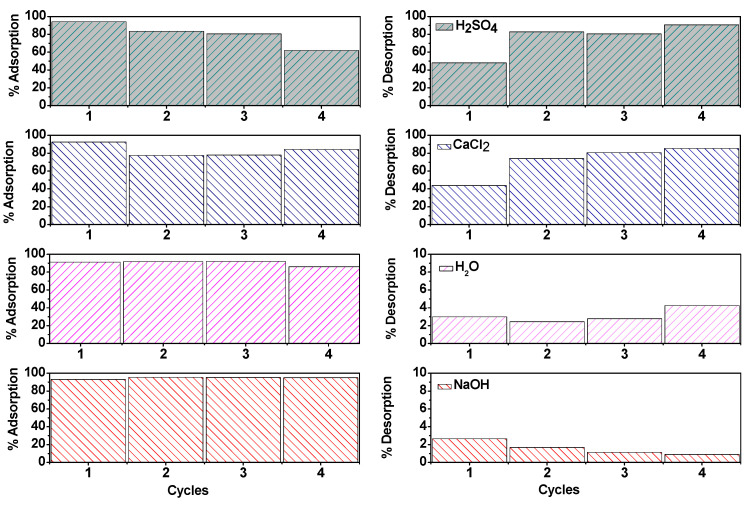
Cycles of adsorption and desorption of MB using A_L_PB and different desorption solutions. The numbers 1–4 represent consecutive adsorption and desorption cycles.

To evaluate the recovery of the biomass (A_L_PB) after MB adsorption, desorption studies were performed using different desorbing solutions (0.1 M H_2_SO_4_, 0.1 M NaOH and 0.2 M CaCl_2_) and deionized water as a control (Figure 7).

The highest percent recovery in the first cycle were observed for 0.1 M H_2_SO_4_ (47.91%) and 0.2 M CaCl_2_ (43.79%), while 0.1 M NaOH showed low desorption efficiency, similar with the control (2.65% and 2.99%, respectively). The increased desorption efficiency with CaCl_2_ is attributed to Ca^2+^ cations competing with MB molecules for binding sites, thereby displacing adsorbed MB molecules [26,47]. Conversely, H_2_SO_4_ promoted desorption by creating acidic conditions, where H^+^ ions replaced MB molecules bound to negatively charged sites [28].

The reusability of the A_L_PB was evaluated over four consecutive adsorption–desorption cycles using the same desorbing solutions. When 0.1 M NaOH and control were used as desorbing solutions, the adsorption and desorption percentages remained similar across cycles. In contrast, when 0.1 M of H_2_SO_4_ and 0.2 M CaCl_2_ were used, the adsorption capacity decreased by nearly 15% after the third cycle, while desorption efficiency increased from 47.91% to 80.48% for 0.1 M H_2_SO_4_ and from 43.79% to 80.70% for 0.2 M CaCl_2_.

These results suggest that acid and high-ionic-strength solutions progressively enhance MB desorption but can also alter the structural integrity of the biomass or compete for the active binding sites. As discussed in previous sections, strong acids such as H_2_SO_4_ can partially degrade polysaccharides and proteins, leading to modification in functional groups, reduced porosity, and, consequently, limited access to active sites to further MB adsorption. Meanwhile, during consecutive cycles, Ca^2+^ ions can form electrostatic interactions with carboxylate and sulfonate functional groups, blocking binding sites and reducing their ability to interact with MB in later cycles [17].

### 2.5. Practical Application

The adsorption efficiency of acid- or alkali-pretreated *M. pyrifera* can be affected by different factors, including the charge properties of various pollutants (e.g., neutral, anionic, or cationic charge), the presence of competing ions, and the physicochemical and biological properties of domestic or industrial wastewater, among others [47,48,49]. Therefore, for practical applications, the influence of all these factors needs to be considered.

As a first approach, in this study, MB cationic dye was used as a model molecule of organic pollutants, showing promising results in adsorption, mainly due to the electrostatic attraction between the negative charge of the surface functional groups of alkali-pretreated *M. pyrifera* and the positive charge of the cationic dye. However, the adsorption efficiency can be different in the presence of anionic, neutral, or a mixture of contaminants with different charges, which can decrease the adsorption efficiency due to electrostatic repulsion or a weaker interaction mechanism [48,50].

Additionally, future research is necessary to evaluate the selectivity and adaptability of alkali-pretreated *M. pyrifera* in real wastewater systems, including industrial and domestic effluents. Under real conditions, the pH variations, the presence of dissolved organic matter, and the coexistence of a wide range of pollutants, among others, have a significant influence on the adsorption process [51,52], whereas in the literature, this type of study has been more limited.

The comparison of the performance of the seaweed-based biosorbent with conventional adsorbents (e.g., activated carbon), considering biomass regeneration and reuse, cost–benefit analysis, and integration into existing wastewater treatment plants, is also necessary for large-scale implementation. This assessment should also consider exploring more friendly regeneration methods for the biosorbent to minimize chemical waste and improve the feasibility of large-scale applications. In this sense, an interesting approach is an assessment through the Life Cycle Assessment (LCA) to evaluate the environmental impact of the use of alkali-pretreated *M. pyrifera* for organic pollutants removal and to identify the “host points” throughout all stages, from the preparation of the adsorbents to their regeneration, reuse, and subsequent disposal.

Overcoming these challenges will be essential for advancing seaweed-based biosorption from a promising laboratory technique to a viable and sustainable solution for industrial wastewater treatment.

## 3. Materials and Methods

### 3.1. Chemicals

Methylene blue (C_16_H_18_CIN_3_S, analytical grade), sulfuric acid (H_2_SO_4_, 97%), sodium hydroxide (NaOH, ≥99%), calcium chloride dihydrate (CaCl_2_·2H_2_O, ≥98%), and sodium chloride (NaCl, ≥99%) were supplied by Merck (Darmstadt, Germany). All chemicals were used as received.

### 3.2. Biosorbent Preparation

*Macrocystis pyrifera* biomass was obtained from two management and exploitation areas for benthic resources (MEABRs) located in the Valparaíso Region, central Chile (32°38′ S 71°26′ W; 32°42′ S 71°29′ W). The biomass was washed thoroughly using deionized water and oven-dried at 60 °C until a constant weight was reached. The dried biomass was crushed using an analytical mill (Cole Parmer, model 4301-02, Vernon Hills, IL, USA) and stored at room temperature until needed. The biomass was pretreated with acid or alkali treatment, according to Ata et al. [16]. Briefly, 10 g of biomass was mixed with 1000 mL of 0.1 M H_2_SO_4_ or 0.1 M NaOH for acid and alkali treatment, respectively. The resultant acid and alkaline solutions were stirred at 100 rpm for 24 h. At the end of the treatment, the biosorbents were centrifuged at 5000 rpm for 10 min, washed thoroughly with deionized water, and dried in an oven at 60 °C for 6 h. The biosorbents obtained were named as follows: untreated biomass (UB), acid-pretreated biomass (A_C_PB), and alkali-pretreated biomass (A_L_PB). The experiments were performed in duplicate.

### 3.3. Biosorbent Characterization

UB, A_C_PB, and A_L_PB were characterized by Fourier transform infrared spectroscopy (FTIR-ATR), textural properties, scanning electronic microscopy (SEM), and potentiometric titrations.

The Fourier transform infrared spectra (FTIR) were recorded on a FTIR-ATR spectrometer (Bruker, ALPHA II, Karlsruhe, Germany) over a wavenumber range of 400–4000 cm^−1^. Textural properties were determined from N_2_ adsorption-desorption isotherms at 77 K with 3-Flex Micromeritics equipment. Before the analysis, the samples were degassed for 4 h at 573 K using a SmartVacPrep device (Micromeritics, Norcross, GA, USA). The Brunauer, Emmett, and Teller (BET) method [53] was used to calculate the specific surface area while that pore size distribution was determined by the Barrett–Joyner–Halenda (BJH) method [54] and the total pore volume resulting from the adsorbed quantity at p/p_0_ of 0.99. The morphologies of the biomass were determined by scanning electron microscopy (SEM) using a Tescan, Vega3, Brno, Czech Republic, instrument on gold-palladium coated biomass.

Potentiometric titrations were performed at 20 ± 0.5 °C under a nitrogen atmosphere to avoid carbon dioxide dissolution into the solution. A biomass concentration of 1.0 g L^−1^ of UB, A_C_PB, or A_L_PB was suspended in 100 mL of NaCl solutions at different ionic strengths (0.001 M, 0.01 M, and 0.1 M) for zero-point charge determination (PZC), with stirring maintained until the pH remained constant. Titrations were initiated from the natural pH of the suspensions by adding 0.2 mL of either HCl (0.1 M) or NaOH (0.1 M), allowing the pH to stabilize before each subsequent addition. The pH response of electrodes was calibrated with buffer solutions at 4.0, 7.0 and 10. The PZC was determined from the intersection point of the potentiometric titration’s curves obtained at different ionic strengths. Meanwhile, intrinsic acid dissociation constants (pka_1_, pka_2_, and pka_3_) were determined using the simple extrapolation method, based exclusively on using titration data obtained at 0.1 M NaCl [39,55]. All assays were performed in duplicate to ensure reproducibility.

### 3.4. Adsorption and Desorption Tests

#### 3.4.1. Batch Adsorption Studies

The MB biosorption capacity of *M. pyrifera* was determined by introducing 50 mg of biomass (dry weight, *W*) into a conical flask containing 50 mL (*V*) of MB at the desired initial concentration (*C_i_*, mg L^−1^). The sorption process was performed at different initial pHs (3.0, 7.9, and 10), room temperature, and 100 rpm shaking rate for 2 h. When the sorption equilibrium was reached, the solution was separated from the biomass by centrifugation at 3000 rpm for 10 min and analyzed for equilibrium MB concentration (*C_e_*, mg L^−1^) using a UV/VIS spectrophotometer (Quimis, Sao Paulo, Brazil) at 664 nm. All experiments were performed in duplicate and reported as the mean value.

The equilibrium adsorption capacity (*q_e_*), which represents the amount of MB adsorbed by mass unity of *M. pyrifera*, was determined using Equation (1).

(1) $$q_{e}=\frac{\left(C_{i}-C_{e}\right)V}{W}$$

where *V*, volume, is expressed in liters and *W* represents the algae biomass reported in g.

#### 3.4.2. Batch Desorption Studies and Reusability

For the adsorption-desorption studies, 200 mg of A_L_PB was added to 50 mL of MB (200 mg L^−1^) solution. The mixture was agitated at room temperature in a shaker (100 rpm) for 2 h. When the equilibrium time was achieved, the solution was separated from the biomass by centrifugation (at 3000 rpm for 10 min) and the MB concentration was analyzed using a UV/Vis spectrophotometer (Quimis, Sao Paulo, Brazil) at 664 nm. After the biosorption assays, the biomass was washed three times with distilled water to eliminate weakly adsorbed MB molecules. The desorption process was performed with 50 mL of different desorbing solutions (0.1 M H_2_SO_4_, 0.1 M NaOH, 0.2 M CaCl_2_, and distilled water). The suspension was shaken for 24 h, separated by centrifugation, and the solution was analyzed to determine MB concentration. The percent desorption recovery was determined using Equation (2). The reusability was determined by four successive cycles of adsorption-desorption of MB using the different desorbing solutions.

(2) $$\%\;recovery=\frac{concentration\;of\;MB\;desorbed}{concentration\;of\;MB\;\;sorbed}\times 100$$

#### 3.4.3. Equilibrium Modeling

The experimental data were analyzed using different sorption isotherm models: Langmuir [56], Freundlich [57], and Dubinin and Radushkevich (D-R) [58]. The Langmuir isotherm was defined for monolayer adsorption, and there was no interaction between molecules adsorbed on neighboring sites. The linearized form of Langmuir isotherm is given in Equation (3) [59].

(3) $$\frac{C_{e}}{q_{e}}=\frac{1}{Q_{0}K_{L}}+\frac{1}{Q_{0}}C_{e}$$

where *Q*_0_ (mg g_ads_^−1^) is the maximum adsorption capacity and *K_L_* is the Langmuir constant related to adsorption capacity.

The separation factor (*R_L_*) was calculated according to Equation (4), where 0 < *R_L_* < 1, indicates favorable adsorption, *R_L_* = 1 corresponds to linear adsorption, *R_L_* > 1 represents unfavorable adsorption, and *R_L_* = 0 denotes irreversible adsorption [59].

(4) $$R_{L}=\frac{1}{1+K_{L}C_{0}}$$

When the heterogeneity of the sites is very large, the experimental data are usually better represented by a Freundlich isotherm. The linearized form of this isotherm is described using Equation (5) [57,60].

(5) $$lnq_{e}=lnk_{F}+\frac{1}{n}lnC_{e}$$

where *k_F_* is a constant related to the biosorption capacity and *1*/*n* is an empirical parameter related to the biosorption intensity, which varies with the heterogeneity of the material.

Finally, the Dubinin–Radushkevich (D-R) model can be described using Equation (6). The D-R model determines the nature of the biosorption process as physical or chemical, where the linear representation of the D-R isotherm equation is presented by Equation (6) [17].

(6) $$lnq_{e}=lnq_{m}-\beta \varepsilon^{2}$$

where *q_m_* is the maximum adsorption capacity (mol g_ads_^−1^), *β* is the activity coefficient related to mean free adsorption energy (mol^2^ kJ^−2^), and *ε* is the Polanyi potential. The values of *q_m_* and *β* can be determined from the slope and intercept of a lineal *ln q_e_* vs. *ε*^2^ plot. The *ε* value is calculated using Equation (7).

(7) $$\varepsilon =RTln\left(1+\frac{1}{C_{e}}\right)$$

where *R* is the gas constant in kJ (mol K)^−1^ and *T* is the absolute temperature (K); the mean free adsorption energy (*E*, kJ mol^−1^) is estimated using Equation (8).

(8) $$Ε=\frac{1}{\sqrt{2\beta }}$$

The mean free adsorption energy can be used to provide information about the mechanisms of the adsorption process. If *E* > 16 kJ mol^−1^, the adsorption is typically chemical, which involves stronger interactions, like covalent bonding. When the *E* values are between 8 and 16 kJ mol^−1^, the adsorption process can be considered as a mix of physical and chemical adsorption, leading toward chemical adsorption, whereas if *E* is lower than 8 kJ mol^−1^, the process is typically physical, which involves weaker forces like van der Waals interactions [61].

### 3.5. Sorption Kinetics Studies

Sorption kinetics of MB using alkali-pretreated biomass were performed using 100 mL of MB (200 mg L^−1^) at room temperature and different initial pHs (3.0, 7.9, and 10) and biosorbent dosages (1.0–4.0 g L^−1^). Samples of 1 mL were withdrawn each time for 2 h and analyzed for MB concentration using a UV/VIS spectrophotometer (Quimis, Sao Paulo, Brazil) at 664 nm.

To study the mechanism of biosorption of MB on *M. pyrifera* pretreated with alkali, the experimental data were adjusted to the pseudo-first-order kinetic Lagergren model [62], the pseudo-second-order model [63], liquid-film diffusion [64], and intra-particle diffusion model [65].

The pseudo-first-order kinetic Lagergren model can be expressed as an integrated rate Equation (9), considering the boundary conditions *t* = 0 to *t* and *q_t_* = 0 to *q_e_* [59].

(9) $$log\left(q_{e}-q_{t}\right)=log\;q_{e}-\frac{k_{1}}{2.303}t$$

where *k*_1_ (min^−1^) corresponds to the pseudo-first-order sorption rate constant, *q_e_* is the amount of solute adsorbed on the surface at equilibrium, and *q_t_* is the amount of solute adsorbed at any time; as described above, *q* values are expressed in mg g_ads_^−1^. A straight line in a *log (q_e_ − q_t_)* versus *t* plot gives (*−k*_1_/2.303) the intercept as slope and *log q_e_.*

The pseudo-second-order kinetic model is described by Equation (10), where *k*_2_ represents the pseudo-second-order sorption rate constant, determined from the plot of *t/q* versus *t* [59,63].

(10) $$\frac{t}{q}=\frac{1}{k_{2}q_{e}^{2}}+\frac{t}{q_{e}}$$

According to the model of Boyd et al. [64], the liquid-film diffusion rate constant (*k_fd_*. min^−1^) was calculated from the slop of the plot of *Ln*(1 − *F)* versus *t* (Equation (11)).

(11) $$-ln\left(1-F\right)=k_{fd}\;t$$

*F* is the fractional attainment (*F* = *q_t_/q_e_*) at time *t*.

According to the model developed by Weber and Morris [65], the intra-particle diffusion rate constant, *k_i_* (mg g_ads_^−1^ min^1/2^) and the *C_i_* constant (mg g_ads_^−1^), can be evaluated from the slope and intercept of *q_t_* vs. *t*^0.5^ plot, respectively, using Equation (12) [66].

(12) $$q_{t}=k_{i}t^{0.5}+C_{i}$$

## 4. Conclusions

In this work, it has been demonstrated that the physicochemical and structural properties of acid and alkali pretreatment of *M. pyrifera* affect the MB adsorption. Alkali pretreatment improved the maximum adsorption capacity of MB by 58%, while acid pretreatment decreased MB adsorption by 43% compared with untreated biomass. The enhanced MB adsorption of alkali pretreatment was due to increased surface porosity and higher exposure of hydroxyl, carboxylate, and sulfonate functional groups, as was confirmed by FTIR-ATR and potentiometric titrations. In contrast, the acid pretreatment induced a more compact seaweed structure with a higher exposition of protonated functional groups, such as carboxylic, amine, and amide, which reduced the electrostatic attraction with the MB cationic dye.

The adsorption process followed the Langmuir isotherm model, suggesting mono-layer adsorption, whereas the pseudo-second-order kinetic model suggested a chemisorption process. According to the energy values (8.45–10 kJ mol^−1^), the primary adsorption mechanism was the electrostatic interaction between the negative surface functional groups in alkali *M. pyrifera* with the positive charges of MB. According to the intra-particle diffusion model, the biosorption occurs through a two-step adsorption process, where MB first adsorbs onto the seaweed, followed by diffusion into internal pores. In addition, according to our results, successive cycles could be used without loss of removal efficiency. Finally, we conclude that *M. pyrifera* pretreated with alkali has significant potential to be used as a biosorbent for removing organic pollutants from aquatic systems. Future research should evaluate the performance of acid- or alkali-pretreated *M. pyrifera* in more complex wastewater, along with its economic feasibility and sustainable regeneration strategies, including more sustainability pretreatments and biomass reuse, to advance its practical application in biosorption technologies.

## Figures and Tables

**Table 1:** Molecular characteristics of methylene blue [29].

Molecular Characteristics	
Chemical formula	C_16_H_18_ClN_3_S
Molecular weight (g mol^−1^)	319.9
Solubility in water (g L^−1^)	50
pKa	3.8

**Table 2:** Surface characterization of untreated (UB), acid-pretreated (A_C_PB), and alkali-pretreated (A_L_PB) seaweed biomass.

Biomass	Specific Surface Area m^2^ g^−1^	Pore Volume cm^3^ g^−1^	Pore Diameter nm
UB	0.5	0.0021	114
A_C_PB	0.3	0.0012	87
A_L_PB	0.8	0.0065	86

**Table 3:** PZC and intrinsic acid dissociation constant (pka) determined by potentiometric titration.

Biomass	PZC	Intrinsic pKa	Acid Groups [26]
UB	7.1	pKa_1_ = 5.64	Carboxylic
		pKa_2_ = 8.20	Amine
		pKa_3_ = 9.33	Amino
A_C_PB	5.1	pKa_1_ = 4.24	Carboxylic
		pKa_2_ = 6.38	Phosphonate
		pKa_3_ = 9.02	Amino
A_L_PB	5.4	pKa_1_ = 5.82	Carboxylic
		pKa_2_ = 10.82	Phenolic, sulfhydryl
		pKa_3_ = 11.1	sulfhydryl

**Table 4:** Constants of Langmuir and Freundlich models for untreated biomass (UB), acid-pretreated biomass (A_C_PB), and alkali-pretreated biomass (A_L_PB) performed at room temperature, 1.0 g L^−1^ of biosorbent dosage, and pH 7.9.

Biomass	Langmuir Constants				Freundlich Constants			Adsorption Energy ^1^	
	*Q*_0_ mg g^−1^	*K*_1_	R^2^	*R_L_*	*n_F_*	*K_F_*	R^2^	*E* kJ mol^−1^	R^2^
UB	333	0.032	0.98	0.050	1.561	14.450	0.84	8.45	0.93
A_C_PB	189	0.021	0.99	0.074	0.527	74.896	0.96	9.13	0.97
A_L_PB	526	0.036	0.99	0.034	1.906	36.778	0.83	10.00	0.88

^1^ Determined by Dubinin–Radunshkevich model (D-R).

**Table 5:** Comparison of maximum adsorption capacity of MB on several adsorbents.

Adsorbent	Biosorbent Dose (g/L)	pH	Contact Time (min)	*Q*_0_ (mg g^−1^)	References
Untreated *M. pyrifera*	1.00	7.9	120	333	This study
Acid-pretreated *M. pyrifera*	1.00	7.9		189	This study
Alkali-pretreated *M. pyrifera*	1.00	7.9		526	This study
*Nizamuddinia zanardinii*	0.24	6.5	180	142.08	[5]
Alkali *Nizamuddinia zanardinii*	0.24	6.5		99.69	[5]
Untreated *Gracilaria parvispora*	0.24	6.5		87.78	[5]
Alkali *Gracilaria parvispora*	0.24	6.5		110.64	[5]
Untreated *Ulva fasciata*	0.24	6.5		143.97	[5]
Alkali *Ulva fasciata*	0.24	6.5		149.28	[5]
*Algae gelidium*	NR	6.0	180	171.00	[44]
*Sargassum muticum* raw biomass	0.20	7.0	120	142.87	[35]
Biochar from municipal sludge and tea waste	10.0	7.0	1440	12.57	[37]

NR: not reported.

**Table 6:** Kinetic constants for MB adsorption on *M. Pryrifera* pretreated with alkali solution at different pHs and biosorbent dosages.

Biomass	pH 3.0			pH 7.9			pH 11		
	A_L_PB Dosage (g L^−1^)			A_L_PB Dosage (g L^−1^)			A_L_PB Dosage (g L^−1^)		
	1.0	2.0	4.0	1.0	2.0	4.0	1.0	2.0	4.0
Pseudo first order model									
*q_exp_* (mg g^−1^)	156	92	47	185	96	50	188	97	49
*q* (mg g^−1^)	75	48	24	60	25	5	70	27	10
*k*_1_ (min^−1^)	0.03	0.03	0.04	0.03	0.03	0.05	0.03	0.04	0.03
R^2^	0.75	0.86	0.92	0.78	0.73	0.64	0.81	0.84	0.70
Pseudo second order									
*q* (mg g^−1^)	164	96	48	189	97	50	192	97	49
*K*_2_ (g mg^−1^ min^−1^)	0.0007	0.0014	0.0039	0.0021	0.0057	0.0680	0.0015	0.0079	0.0159
R^2^	0.99	0.99	0.99	0.99	0.99	1.00	0.99	0.99	0.99
External mass transfer (Liquid film diffusion)									
*K_fd_*	0.0297	0.0337	0.0385	0.0324	0.0324	0.0491	0.0342	0.0586	0.0332
R^2^	0.752	0.857	0.915	0.783	0.726	0.637	0.812	0.753	0.7032
Intra-particle diffusion									
*k_dif_* (mg g^−1^ min^−1/2^)	25.14	14.82	7.89	33.41	17.76	9.64	33.20	18.07	9.194
R^2^	0.99	0.99	0.99	0.96	0.94	0.93	0.98	0.92	0.93
*k_dif_* (mg g^−1^ min^−1/2^)	1.572	1.063	0.488	1.297	0.525	0.0211	1.334	0.558	0.196

## Data Availability

Data are contained within the article and Supplementary Materials.

## Supplementary Materials

The following supporting information can be downloaded at: https://www.mdpi.com/article/10.3390/ijms26073307/s1.
