# Supplementary material for: Enhancing the Biosorption Capacity of Macrocystis pyrifera: Effects of Acid and Alkali Pretreatments on Recalcitrant Organic Pollutants Removal
Source: Int J Mol Sci. 2025 Apr 2;26(7):3307. doi: 10.3390/ijms26073307 (PMC11989721; doi:10.3390/ijms26073307)
Supplement: Supplementary file 1 [file ijms-26-03307-s001.zip › ijms-3477065-supplementary.pdf]

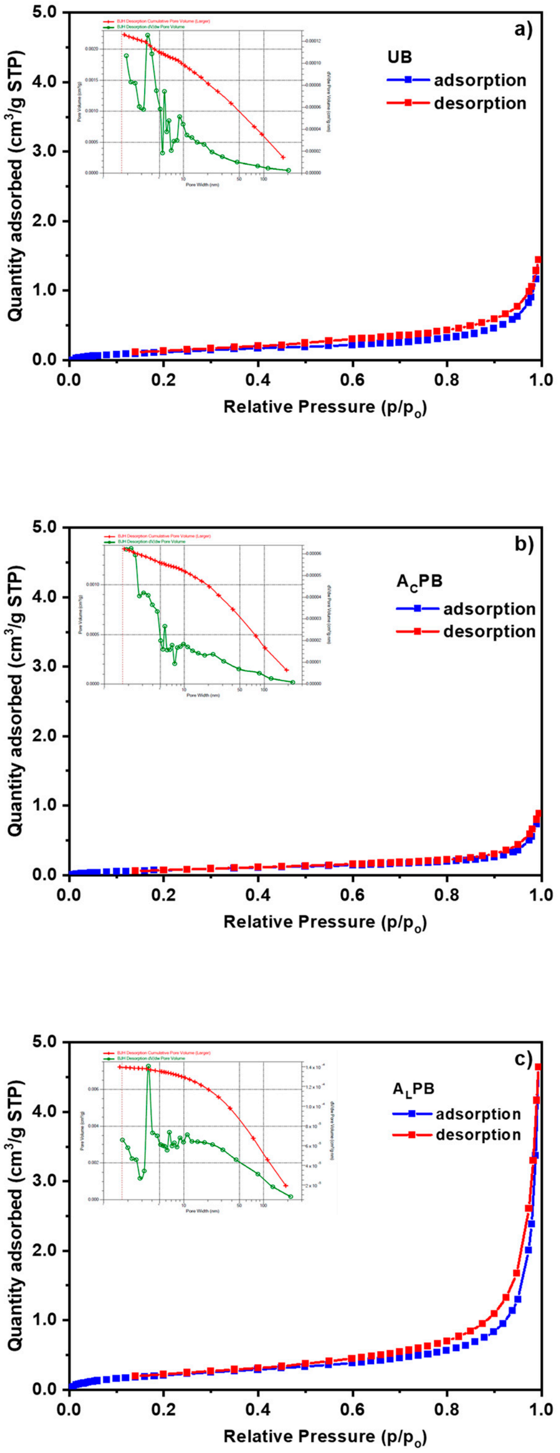

Figure S1. N_2 adsorption-desorption isotherms (a) untreated *M. Pyrifera* (b) acid pretreatment and (c) alkali pretreatment. Inset: corresponding BJH pore size distribution of the biosorbents.

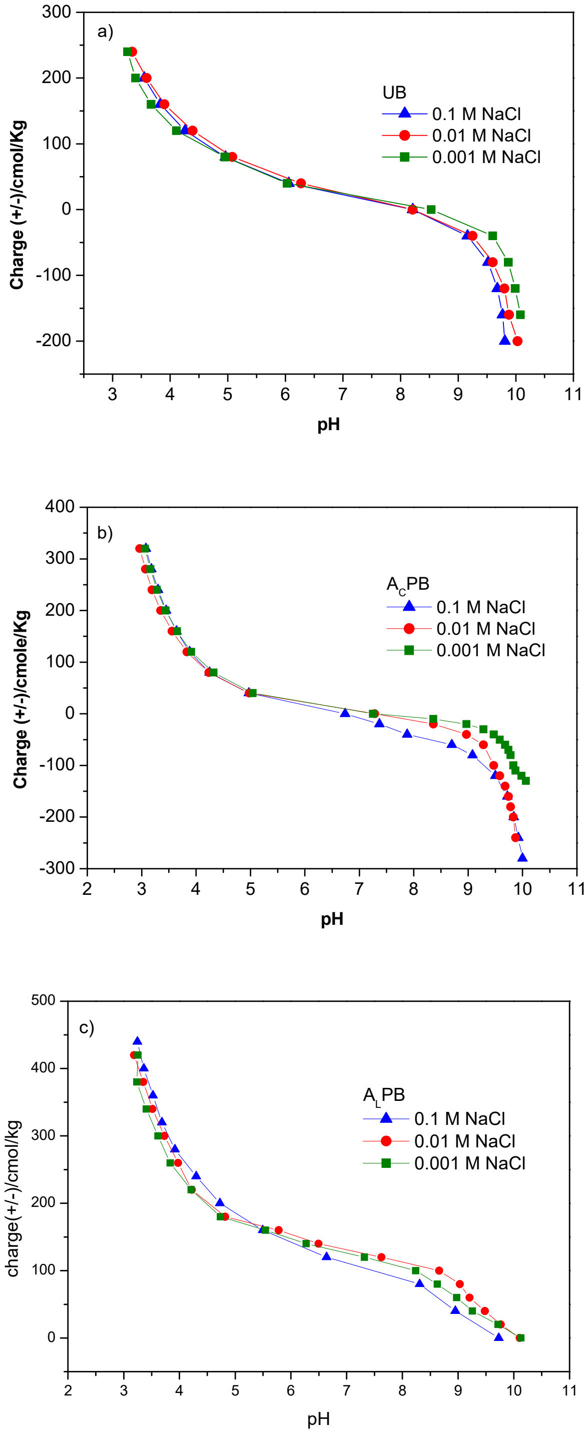

Figure S2. Graphic determination of the PZC with three ionic strengths (a) untreated *M. Pyrifera* (b) acid pretreatment and (c) alkali pretreatment.
